# Intestinal Barrier Dysfunction and Microbiota–Gut–Brain Axis: Possible Implications in the Pathogenesis and Treatment of Autism Spectrum Disorder

**DOI:** 10.3390/nu15071620

**Published:** 2023-03-27

**Authors:** Vanessa Nadia Dargenio, Costantino Dargenio, Stefania Castellaneta, Andrea De Giacomo, Marianna Laguardia, Federico Schettini, Ruggiero Francavilla, Fernanda Cristofori

**Affiliations:** 1 Interdisciplinary Department of Medicine, Pediatric Section, Children’s Hospital ‘Giovanni XXIII’, University of Bari “Aldo Moro”, 70126 Bari, Italy; 2 Child Neuropsychiatry Unit, Department of Translational Biomedicine and Neuroscience, University of Bari “Aldo Moro”, 70126 Bari, Italy; 3 Neonatology and Neonatal Intensive Care Unit (NICU), University of Bari “Aldo Moro”, 70126 Bari, Italy

**Keywords:** autism spectrum disorder, intestinal barrier dysfunction, leaky gut, microbiota–gut–brain axis, probiotics, children

## Abstract

Autism spectrum disorder (ASD) is a complex neurodevelopmental disorder with multifactorial etiology, characterized by impairment in two main functional areas: (1) communication and social interactions, and (2) skills, interests and activities. ASD patients often suffer from gastrointestinal symptoms associated with dysbiotic states and a “leaky gut.” A key role in the pathogenesis of ASD has been attributed to the gut microbiota, as it influences central nervous system development and neuropsychological and gastrointestinal homeostasis through the microbiota–gut–brain axis. A state of dysbiosis with a reduction in the *Bacteroidetes*/*Firmicutes* ratio and *Bacteroidetes* level and other imbalances is common in ASD. In recent decades, many authors have tried to study and identify the microbial signature of ASD through in vivo and ex vivo studies. In this regard, the advent of metabolomics has also been of great help. Based on these data, several therapeutic strategies, primarily the use of probiotics, are investigated to improve the symptoms of ASD through the modulation of the microbiota. However, although the results are promising, the heterogeneity of the studies precludes concrete evidence. The aim of this review is to explore the role of intestinal barrier dysfunction, the gut–brain axis and microbiota alterations in ASD and the possible role of probiotic supplementation in these patients.

## 1. Introduction

### 1.1. Autism Spectrum Disorder

ASD is a complex neurodevelopmental disorder with multifactorial etiology, characterized by the impairment of two main functional areas: (1) continuous communication and social interaction deficits, and (2) restrictive and repetitive behaviors and interests. The incidence among sexes is a male/female rate of 3:1 [1,2].

ASD diagnostic criteria are available in the Diagnostic and Statistical Manual of Mental Disorders-5 [3], describing persistent deficits in social communication and social interaction across multiple contexts, such as deficits in social–emotional reciprocity; nonverbal communicative behaviors; and developing, maintaining and understanding relationships. Moreover, ASD children present behavior restricted and repetitive patterns of interests or activities, as manifested by at least two of the following: (a) repetitive or stereotyped motor movements, use of objects or speech; (b) insistence on sameness, inflexible adherence to routines or ritualized patterns of verbal or nonverbal behavior; (c) fixated and restricted interests that are abnormal in intensity or focus; and (d) hyper- or hypo-reactivity to sensory input or unusual interest in sensory aspects of the environment. Such symptoms must be present in the early period of development, be generally evident at the age of 3 and cause significant clinical impairment of global functioning. In 2014 in the United States, the Centers for Disease Control (CDC) estimated 1 case out of 68 children of the age of 8 [4], and in 2018, the CDC reported an ASD prevalence rate of 1 in 44 or an incidence rate of 2.3% [5].

Considering the great geographical variability and the methodologic differences employed in prevalence studies, the actual prevalence of ASD is estimated to be higher than 2.5% in the United States and 1.5% in Denmark, Finland and Sweden. In Italy, the prevalence of children diagnosed with ASD is estimated to be 1 out of 77 among children between 9 and 17 years old, with a higher risk for the male sex; the number of male patients is four times higher than the number of female patients.

### 1.2. ASD Etiopathogenesis

The disease is now defined as a complex disorder with multifactorial etiopathology because it seems to be determined by the contribution of many risk factors, such as genetics, epigenetics and environmental factors.

Much evidence supports genetic factors as the predominant cause of ASD. Emphasis on the genetic component from epidemiological data derives from familiarity and the high incidence of the autistic behavioral phenotype in the context of genetic diseases with well-known etiology. The amount of ASD cases with a genetic base represents 10–20% of the overall number of autistic patients. Concordance studies on twins have shown very suggestive data; the concordance among monozygotic twins has been shown to be variable from 70% to 90%, whereas that between dizygotic twins is between 0–10% [6]. Moreover, ASD incidence is estimated to be 2% among siblings, with a 100 times higher risk than the normal population (0.02%). Furthermore, the recurrence risk is significantly higher in families with a first diagnosis of ASD than it is in the normal population. In particular, families with a first child diagnosed with ASD have greater probabilities of having an autistic second child depending on the child’s sex, as follows: 15–25% if the child is male, and 5–15% if the child is female. Last, the presence of many first-grade relatives diagnosed with ASD testifies to the importance of the genetic hypothesis [7,8]. There are ASD forms that are similar to genetic syndromes, and these forms represent 10% of all ASD diagnoses. Beyond the findings of specific alterations that cause specific syndromes, genetic anomalies implied in ASD can be caused by mutations in one gene or in the total number of copy variations (CNVs). Besides specific genetic alterations that cause well-known clinical pictures, there are uncommon genetic variants (with a documented presence of less than 1% of the general population). These mutation sites are in correspondence with genes of great importance in the process of neurodevelopment, and the most documented are CNVs, such as microdeletion or microduplication; nonsense mutation with the insertion of a stop codon; and missense mutation with the creation of aberrant products, such as inactive proteins or proteins with reduced biological activity [9].

Some relevant examples include genes that code for synaptic transmembrane proteins, such us neuroligine 3 and 4 and neurexine 1 and 3, which are crucial for synaptic function; others are SHANK family genes (SHANK 1, 2, 3) that codify for proteins involved in synapsis formation and dendritic spine maturation. In detail, SHANK 3 is involved in dendritic development and in a pathway with reelin, a protein that is essential for the stabilization and the laminar organization of the cerebral cortex [10]. Even if the scientific community well accepts the role of genetic anomalies, many studies have shown similar associations with environmental risk factors.

A plethora of environmental risk factors have been taken into consideration, and most of them refer to the pre/peri-natal period because the maximum development of the central nervous system (CNS) is in this period. Risk factors that influence neurodevelopment and provoke long-term alterations in the brain’s physiology include pre-natal exposition to viral infections (e.g., Cytomegalovirus and Rubivirus); environmental toxic substances, such as pesticides, phthalates, solvents, environmental pollutants, and heavy metals; stress; alcohol intake; and diet [11]. An association between maternal conditions and ASD risk has been demonstrated.

### 1.3. Physiological Aspects of ASD

The most common predisposing factors of ASD are neurodevelopment anomalies during the first and the second trimester of pre-natal life [12]. Other causes are less frequent but not completely negligible. Among these, cerebellar damage has been identified in the peri-natal period, which increases the risk of developing autism by 30 times. Neuropathology post-mortem studies on histological samples of CNS taken from autistic patients have shown the presence of cytoarchitectonic anomalies, which can involve various brain regions. Among these, reduced apoptosis and/or enhanced cellular proliferation (particularly evident in macrocephalic patients), neuronal migration alteration or anomalies in the process of cellular maturation and differentiation have been described [13]. From a functional point of view, neuropathological anomalies bring the formation of an atypical neural network characterized by reduced long-distance connectivity and exceeding local connectivity [14].

### 1.4. Gastrointestinal Involvement in ASD

A bidirectional interaction between the gastrointestinal (GI) tract, gut microenvironment and CNS, called the ‘microbiota–gut–brain axis’, regulates intestinal and neurological homeostasis. An impairment of this complex system can promote, in the presence of other contributing factors, the pathogenesis of nervous-system-related diseases, such as ASD.

Enteric symptoms (including constipation, diarrhea, recurrent abdominal pain/bloating and gastroesophageal reflux) are frequent among ASD patients, who often present alterations in intestinal motility and dysfunction of the epithelial barrier.

The aim of this review is to explore the current scientific evidence on the role of intestinal barrier dysfunction, the gut–brain axis and microbiota alteration in ASD and the possibility of modulating gut microbiota (GM) in these patients using probiotic supplementation.

## 2. Intestinal Permeability in ASD

Children with ASD frequently exhibit GI tract problem symptoms. These illnesses’ underlying causes, though, are still poorly understood. It is speculated that the pathophysiology of ASD may be influenced by the GM and its metabolites [15,16,17]. Numerous articles have identified the impact of GI alteration, GM and CNS function, as well as the potential participation of the microbiome–gut–brain axis [18]. Given that the prevalence of GI symptoms in ASD children may reach 70%, microbiome and gut–brain connections are likely to play a significant role in ASD [19].

Additionally, the severity of ASD is correlated with the prevalence of GI symptoms [20], demonstrating the role of the gut in the pathogenesis of ASD [21,22]. ‘Leaky Gut syndrome’ refers to a situation in which the small or large intestine’s epithelial barrier function is compromised, resulting in increased types and quantities of molecules and cells that can flow from the gut to the circulatory system and vice versa [23].

The intestinal microbiota, mucous layer, intestinal epithelium, elements of innate and acquired immunity, hormonal and neuroenteric systems, vascular–lymphatic system and digestive enzymes comprise the functional unit of intestinal permeability. It serves as the body’s first line of protection against toxic, immunogenic and pro-inflammatory substances by maintaining a delicate balance between the intestinal lumen’s antigenic charge and the intricate structure of the intestinal mucosa. It is crucial for preserving good health, stopping systemic and intestinal inflammation and suppressing the immune system. Only trace amounts of antigens can pass through the normal intestinal barrier to engage with the innate and adaptive immune systems. A change in its function may promote bacterial and antigen transit, which may then result in pathogenic diseases [24]. The core of intestinal permeability is the occlusive, tough intracellular connections. These tight junctions comprise a system of numerous proteins in the paracellular space between each cell in the gut lining. They are indeed responsible for the epithelial barrier’s functionality. This insurmountable but selective barrier is reinforced by a thick mucus coating and interacts steadily with luminal contents and enteric bacteria.

It is well known that the gut and the brain have a strong relationship and regularly interact. Neuropeptides that allow two-way communication between the gut and the brain include substance P, calcitonin gene-related peptide, neuropeptide Y and vasoactive intestinal polypeptide [25]. Cortisol, a key player in developing anxiety and depressive disorders, is also released by the hypothalamic–pituitary–adrenal axis and controls intestinal motility, integrity and hypersecretion [26]. The GM can sequentially affect the function of the CNS through neuronal, endocrine, immunological and metabolic processes because communication is bidirectional [27].

The relationship between the gut and the brain in the etiology of autism is assumed to be increased gut permeability, which has been linked to ASD. For instance, it was demonstrated that the injection of propionic acid (that is produced by intestinal bacteria) in rats’ brains [28] results in neuroinflammation and symptoms resembling those of ASD [29]. This might explain why children with ASD experience worsened symptoms when exposed to food preservatives containing propionic acid. In this particular case, increased gut permeability would allow propionic acid to enter the bloodstream and eventually leak into the blood–brain barrier.

Intestinal permeability prevents intestinal contents from entering the bloodstream and suppressing subsequent immunological inflammatory responses and GI illnesses [30]. As a result, an intact gut barrier decreases inflammatory responses. De Magistris et al. showed that 36.7% of ASD children have aberrant intestinal permeability compared to less than 5% of control children [31]. Similar data were reported by D’Eufemia et al., who reported that 43% of ASD children with GI symptoms have impaired intestinal permeability [32]. Recently, to investigate the association between intestinal permeability and behavior, Teskey et al. measured the intestinal fatty acid binding protein as a marker of intestinal epithelial damage in the plasma of children with ASD and found that an increase in this protein correlates with a severe deficit in communication, social interaction and maladaptive behavior [15].

A broader understanding of the role of the intestinal barrier in autism can greatly help predict the best treatment option for a given patient.

## 3. Microbiota–Gut–Brain Axis Involvement in ASD

Two millennia ago, Hippocrates stated that “All disease begins in the gut” [33]. A growing interest in systems and organs closely related to the CNS has been triggered by the high prevalence of some specific medical comorbidities, such as GI disorders, in individuals with ASD compared to peers with typical development.

Over the past decade, the bidirectional communication between the gut and the brain, the so-called “gut–brain axis,” has been the focus of preclinical and clinical research, investigating its possible role in the etiopathogenesis of some neuropsychiatric conditions, including ASD [34]. This interplay of bidirectional communication connecting mind and body provides a physiological rationale for interpreting these conditions within the biopsychosocial model. The biopsychosocial model examines the reciprocal and complex interactions among biological, psychological and environmental factors contributing to disease [35], mainly due to the growing knowledge of this axis [36].

The enteric nervous system (ENS) is a well-defined entity capable of regulating the intestinal functions of mobility, secretion and mucosal transport entirely autonomously from the CNS [27]. As demonstrated in the animal model, the ENS, even when the gut is entirely denervated by the CNS, it can function on its own. However, it maintains a bidirectional communication pathway with the CNS [27].

The CNS, after integrating a variety of information regarding internal and external environmental changes, performs parasympathetic control through the vagus nerve with cholinergic efferents acting on the myenteric plexus (motor movements) and Meissner’s plexus (secretions of the submucosal glands), and with sympathetic control through the splanchnic nerves that reduce the motility of the intestine and blood supply to the splanchnic circulation [27]. The pronounced synergy and continuous exchange of information along this axis is possible because of the vast neurochemical assets available to the enteric nervous system. In in vivo studies, the peripheral stimulation of vagal fibers has been shown to lead to dopamine release in the reward system [37]. This type of neuronal network, which connects the GI tract with different levels of the CNS involving the aforementioned neural pathways, humoral signaling molecules and hormones [22,38], is the functional basis of the gut–brain axis [39].

Notably, the hypothalamic–pituitary–adrenal axis is also involved in this network. It is responsible for coordinating the body’s adaptive responses following stressful events, modulating the composition of the GM and the integrity of the intestinal barrier through the secretion of norepinephrine and dopamine at the neuroendocrine level. The resulting dysbiosis state and altered permeability can induce the translocation of bacterial components, which promotes the secretion of adrenocorticotropic hormone, corticosterone, prostaglandins and proinflammatory cytokines [26].

Increasing evidence has demonstrated the existence of a complex and still not well-understood two-way connection among the GM, intestine and CNS. In recent years, as microbiological and neuroscientific knowledge has advanced and the role played by the GM in host physiology has become more evident, there has been a shift in the conception of the gut–brain axis, and the term has been renamed the “microbiota–gut–brain axis” [40]. Indeed, communication between gut microbes and the gut–brain axis occurs through multiple pathways and mechanisms, including immune, neural, metabolic and endocrine pathways that arbitrate bidirectional signaling locally in the gut and peripherally [22,41].

Interest in the microbiota–gut–brain axis was ignited when Lozupone et al. demonstrated an increased hypothalamic–pituitary–adrenal axis response to stress in germ-free mice compared with non-germ-free mice [42]. Bravo et al. showed that supplementation with *Lacticaseibacillus rhamnosus* can modify GABA receptor expression in cortical regions, the hippocampus and the amygdala, with subsequent reduction in anxiety- and depression-related behaviors and stress-induced corticosterone levels, suggesting the involvement of the neuroendocrine axis. Interestingly, these effects were reversed after vagotomy, suggesting a crucial role of the peripheral nervous system in the gut–brain connection [43].

Interactions with gut microbes occur in the intestinal barrier, which is an essential and highly dynamic interface between the host and the outside world consisting of several structures, including secretory immunoglobulin (Ig) A molecules, antimicrobial peptides, lysozyme and secretory phospholipase A2, intestinal epithelial cells and adaptive immune cells (macrophages, T cells, B cells and dendritic cells) [22].

Understanding the mechanisms that cause dysfunction in gut–brain communication has made essential contributions to understanding the basic pathophysiology of the microbiota–gut–brain axis in patients with ASD and has encouraged the proposal of new therapeutic perspectives [44].

Indeed, microorganisms harbored in the GI tract can modulate the activation of cells of the immune system and intestinal epithelium, transducing inflammatory or anti-inflammatory signals to the enteric nervous system and, consequently, to the CNS [45]. Several authors have hypothesized that alterations in the GM may contribute to the expression of the autistic phenotype or exacerbate the severity of symptoms in individuals genetically predisposed to ASD [46,47,48]. In fact, the microbiota covers many functions; it supports nutrient digestion, regulates metabolism, processes hazardous substances, participates in detoxification and organizes control of the immune system. Microbiota composition is influenced by many factors, including genes, the maternal microbiome, nutrition, brain activity and mood. This means that something that starts as an emotion in the brain affects the gut and the signals generated by the microbiota. These signals are, in turn, transmitted to the brain, often making that emotional state more intense and prolonged [49].

It has been hypothesized that ASD can result from any disruption that can alter the balance of the microbiota and the gut and that the disruption of a single part of this delicate mechanism can potentially impact any link in the chain [50].

Recent studies have shown that some bacteria belonging to the phylum Bacteroidetes [Barnesiella, Parabacteroides, Bacteroides, Odoribacter, Prevotella, Proteobacteria (e.g., Proteus and Parasutterella) and Alistipes] are more abundant in ASD patients as compared to the general population, whereas Actinobacteria, (Bifidobacterium species) are often less abundant in ASD patients [51,52].

A detailed discussion of microbiota in ASD subjects can be found in Section 4 and Section 5.

Alterations in gut microbial composition can lead to altered levels of neuroactive molecules, such as short-chain fatty acids (SCFAs), particularly propionic acid, acetic acid and butyric acid, and lipopolysaccharides that can induce changes in the CNS through the endocrine pathway [53], and they have all been found to be overexpressed in ASD populations [54], although the results remain mostly contradictory [55,56,57,58].

Studies in animal models have shown an overgrowth of *Firmicutes* species (spp.), a reduction in *Bacteroidetes* spp. and increased butyric acid levels in male mice with autistic-like behaviors [59,60].

Several studies have demonstrated that SCFAs can permeate the blood–brain barrier [61] and modulate the neural characteristics of brain cells [62,63,64]. In fact, the administration of propionic acid in mouse models, pre-natally through a pregnant mother [65,66] and in the early years of life [65,66,67,68], as well as increasing dietary propionic acid in children [69], have facilitated the onset of autism-like behaviors in all animal and human studies.

SCFAs of bacterial species act on specific GI and immune pathways, and this may impair gut metabolic function and increase immune response and mitochondrial dysfunction, resulting in increased oxidative stress. Sustained oxidative stress may, in turn, enhance intestinal permeability and increase inflammation.

Microglial activation in the brain can further increase inflammation, resulting in the malfunction of synapses and manifestation of behavioral abnormalities and neuropathology [70,71,72,73,74].

Like mammalians, also in Drosophila, an alteration in the GM can cause an epithelial oxidative burst, causing changes in gut permeability and affecting longevity and behaviors [75,76,77].

The GM can release metabolites that can modulate levels of psychoactive compounds in the CNS or produce these neuroactive substances on their own [22,78]. Among the different neurotransmitters involved in ASD that appear to be regulated by the microbiome are serotonin, glutamate and dopamine [79,80].

For example, *Bifidobacterium* spp. and *Lactobacillus* spp. are producers of γ-aminobutyric acid (GABA) [79,81,82]; *Candida* spp., *Escherichia* spp., *Enterococcus* spp. and *Streptococcus* spp. are producers of serotonin [81]; *Escherichia* spp. and *Saccharomyces* spp. generate norepinephrine; *Lactobacillus* spp. is a producer of acetylcholine; and *Bacillus* spp. and *Serratia* spp. are producers of dopamine [83]. In addition, elevated levels of norepinephrine have been detected with increased amounts of *Bacillus*, *Enterococcus*, *Escherichia*, *Saccharomyces* or *Streptococcus* spp. in the intestine [79,81].

Serotonin is a neurotransmitter that plays a fundamental role in mood regulation through its influence on microglial cells in the CNS [84]. Enterochromaffin cells distributed along the intestinal mucosa produce 95% of it [85]. In the study by Yano et al., it was shown that indigenous spore-forming bacteria in mouse and human microbiota promote serotonin biosynthesis from enterochromaffin cells and have a significant impact on host physiology by modulating GI motility and platelet function, suggesting direct metabolic signaling from gut microbes to enterochromaffin cells [84]. The GM possesses enzymes that regulate tryptophan metabolism pathways, leading to the production of serotonin, kynurenine or indole derivatives. Therefore, by controlling the amount of tryptophan, the microbiota can influence the brain’s amount of serotonin [86].

Hyper- and hypo-glutamine patterns at different developmental stages underlie the neurotransmitter communication hypothesis in the pathogenesis of ASD. Various studies on the antagonists of NMDARs or AMPARs have shown clinical benefits in ASD [87]. Similarly, the excitatory glutamate pathway is likely one of the factors involved in the etiopathogenesis of ASD. It is part of gut–brain communication, as it mediates trans-synaptic signaling. It is also implicated in cell adhesion, linking pre- and postsynaptic neurons, and it shapes neural networks by specifying synaptic functions [88]. In addition, a correlation between ASD phenotypes and glutamate/glutamine levels in various brain areas has been displayed through the use of in vivo neuroimaging of ASD individuals [87].

Furthermore, altered dopamine signaling has been associated with ASD in both mice and humans. A study by DiCarlo et al. suggested that mice that are homozygous for the T356M DNA variant of the SLC6A3 gene, which encodes the dopamine transporter, manifests altered dopamine signaling and metabolic dysfunction, weigh less and have reduced body fat [89]. The authors found a significant decrease in *Fusobacterium* abundance at the oral level. Moreover, there is a positive association among *Fusobacterium* abundance, better glucose management and decreased body fat [89].

However, neurotransmitters produced in the gut are unlikely to reach the brain because of the presence of the blood–brain barrier. A likely exception is GABA because its transporters are present in the blood–brain barrier. However, the CNS can be indirectly affected by neurotransmitters produced in the gut because they can act on the enteric nervous system [90,91].

In fact, the blood–brain barrier is another crucial anatomo-functional structure of the microbiota–gut–brain axis. Indeed, it modulates the trafficking of specific molecules and contributes to the maintenance of normal neuronal activity. It is also implicated in immunological functions and protects the brain from bacteria and microbial molecules during the CNS developmental phase and in adulthood [92,93]. Balanced GM is necessary to develop and maintain a normal blood–brain barrier.

In addition, structural changes, including increased activation of microglial cells, have been observed post-mortem in the brains of autistic individuals [58,76]. All of this underlies the hypothesis that ASD is a condition caused or at least accompanied by immune activation in the brain that leads to a neuroinflammatory state and could then lead to malfunctioning synapses [76]. In the inflammatory phase, arginine vasopressin is released from the brain, and it is a metabolite known to act on social behavior and is considered a biomarker for ASD [76]. Furthermore, there is a reduced number of Purkinje cells in the cerebellum of ASD patients [35,94], which makes them susceptible to the tetanus neurotoxin produced by *Clostridia tetani* [94]. In fact, a high amount of *Clostridium* spp. was found in subjects with ASD, which could explain the decrease in Purkinje cells in the cerebellum of these subjects [94].

To date, clinical studies investigating neurological function, metabolites related to blood–brain barrier integrity, microbial involvement and GI function in the context of ASD remain lacking and have yet to determine whether they can be exploited as targets for therapies.

## 4. Dysbiosis in ASD Patients

The hypothesis that GI dysbiosis plays a role in the etiopathogenesis of ASD remains a topic as complex as it is crucial. GM changes widely from birth to adulthood, and there is still much debate about the timing of gut bacterial colonization. Recent data suggest that microbes’ initial gut colonization may begin before birth, during pregnancy or through placental colonization [95], although this remains controversial [96,97]. Further colonization begins during and immediately after birth via vaginal delivery (vertical transmission) [98] or, in the case of Cesarean delivery, through contact with the environment immediately afterward (horizontal transmission) [99].

The microbiome fluctuates dynamically as diet and environmental factors change over the course of development [100].

Overall, this transfer of microbial agents to the fetus can help in shaping the immune system, metabolic control and even behavioral aspects [101]. For example, the maternal *Bacteroides* (*B*.) *fragilis* capsular polysaccharide A contributes to the formation of Treg cells in the intestinal mucosa of the fetus, protecting against inflammation and enhancing tolerance to food antigens [102]. Conversely, the composition of the maternal microbiota depends on various influences, such as diet, antibiotic exposure and other environmental factors. A primate study confirmed the role of a high-fat maternal diet in shaping the gut microbiome of offspring [103]. Moreover, maternal consumption of probiotics is correlated with a reduced risk of preterm delivery [104] and the development of allergic diseases in offspring [105]. (On the other hand, antibiotic treatment may limit or alter the fetus’s exposure to bacteria and their products, causing aberrant primitive immunity.)

In mouse models, antibiotic therapy during pregnancy reduces IL-17-producing cells and increases the risk of neonatal sepsis [106]. Hsiao et al. reported that, by stimulating the maternal immune system throughout pregnancy, the offspring show abnormalities in behavior resembling those observed in ASD [107]. Furthermore, by analyzing the offspring’s intestinal microbiota and intestinal mucosa, dysbiosis and increased intestinal permeability were found. However, oral probiotic supplementation with *B. fragilis* during weaning could improve behavioral abnormalities and dysbiosis [107]. Maternal immune activation is also associated with elevated levels of circulating IL-6 and IL-17A [108], which are involved in differentiating Th17 cells. This underlies the hypothesis that the effect of the microbiota on the immune response consists of modulation to maintain a proper balance between pro-inflammatory and anti-inflammatory states mediated by Th17 and Treg cells, respectively [109]. From this, it follows that the GM may be involved in the multifactorial etiology of cognitive and behavioral disorders and protection from them.

Other post-natal factors that shape the gut microbial community include nutritional habits (breast or formula feeding), antibiotic therapy, infections, stress and host genetics [110,111,112].

In general, it has been seen that human breast milk promotes the growth of beneficial bacteria, such as Bifidobacteria [113], which are specialized in the digestion of oligosaccharides contained in human milk [114], with *Bifidobacterium longum* among them [115]. It was found in a study that children with autism experience a significantly shorter breastfeeding period [116]. In addition, a higher risk of developing ASD was observed in children who were not breastfed [76]. Similarly, infants born vaginally have a microbiota with a dominance of *Lactobacillus*, *Prevotella* and *Snethia* spp. [114], whereas those delivered by Cesarean delivery have an altered microbiota composition with a dominance of *Staphylococcus*, *Corynebacterium*, *Propionibacterium* spp. [114,117], *E. coli* and *Clostridium difficile* [118].

In a Swedish study based on a population-based registry with approximately 2.6 million children, elective Cesarean delivery compared with natural vaginal delivery was observed to be associated with a 20% significantly higher risk of developing ASD in the child [119]. On the other hand, no association was reported in the sibling control analysis. Therefore, the authors concluded that Cesarean delivery and ASD are not associated with each other [119]. Similarly, a British study with more than 18,000 participants found no link between Cesarean delivery and ASD [120].

It is an accepted fact that antibiotic use in the first 1000 days of life can have detrimental effects on the normal constitution of the microbiota with long-term effects [121]. It has also been reported that many children with ASD take relatively high doses of oral antibiotics in the first years of life, which could be a factor leading to dysbiosis [122]. It has been observed that even several months after the cessation of antibiotic treatment, recovery of the microbiota to the pre-therapy state is incomplete in a substantial percentage of individuals [123]. Thus, early antibiotic use in infants may alter microbial colonization of the gut and result in decreased taxonomic diversity [123].

The hypothesis about the possible role of the GM in the pathogenesis of autism began with the consideration of *Clostridium* and the hypothesis that the neurotoxic effects of *Clostridium* could be linked to the onset of ASD [94]. Several studies have demonstrated an increased abundance of species belonging to Clostridium in the fecal samples of autistic children [75,124]. It has also been hypothesized that subacute and chronic *Clostridium tetani* infection may be responsible for the neurodevelopmental disorder [94]. This has been supported by investigations with promising results on the administration of oral vancomycin, minimally absorbed by the intestinal mucosa and thus specifically targeting the intestinal lumen and its microbiota in ASD [125].

Dysbiosis can also be affected by emotional and physiological stress, as it can alter the pattern of mucus secretion [126], induce a temporary reduction in gastric emptying [127,128] and cause local immune activity by altering intestinal permeability [129]. All these effects can have a severe impact on the proliferation of intestinal microorganisms.

Moreover, to date, the precise relationship between host genetics and microbial dysbiosis in ASD is unknown. The genetic background is quite complex, and more than 1000 point mutations are associated with this disorder [130,131,132]. In a study by Liu et al., there was evidence of how host genetics shape the fecal microbial community [133]. Many ASD-associated mutations occur in genes encoding synaptic cytoplasmic and cell adhesion molecules, scaffolding proteins and various molecules involved in neurotransmission or the regulation of synaptic protein synthesis [132].

For example, a missense mutation in neuroligin 3 protein [134], which regulates dendritic growth and neuronal morphology, has been identified in patients with ASD [135]. Multiple mutations in the gene encoding the synaptic scaffolding protein “SH3 and multiple ankyrin repeat domains 3” (SHANK3) have been found to be associated with ASD [136], altering cortical neuronal connectivity and increasing the risk of ASD diagnosis [137]. SHANK3 mutations alter the enteric nervous system, leading to abnormalities in GI motility and structural alterations in the GI tract [138,139,140].

Other environmental factors that may alter microbial profiles and contribute to the etiology of ASD include several drugs, including the antiepileptic valproate, that alter gut microbial function, including SCFA production [141]. The risk of ASD diagnosis has also been proven to increase in infants born to mothers who are prescribed valproate [142].

In addition, diet plays a key role in determining the composition and function of the gut microbiome [110,143].

ASD children often consume a more selective diet and refuse to eat certain foods, likely because of hyper/hyposensitivity to sensory input associated with ASD [144,145]. Therefore, a selective diet could influence the composition of the GM. Significant changes in the human GM have been demonstrated after eating an all-animal or plant-based diet for only four days [146]. In general, the intake of a diet rich in vegetables, fruits and fiber is associated with increased richness and taxonomic diversity of the GM [147].

On this subject, in recent years, the emergence of metabolomics has made it possible to detect alterations in metabolites in ASD effectively [148], and major metabolomic studies on blood samples are summarized in Table 1. Decreased levels of antioxidants, high levels of bacterial-derived phenolic compounds and a high concentration of SCFAs have been reported to be found in the urine, stools and blood of children with autism [149,150,151,152]. These factors may result in abnormal neuron mitochondrial function, epigenetically regulating ASD-related gene expression [53,153]. In addition, free amino acid levels are much higher in autistic children than they are in controls and correlate with increased proteolytic bacteria, such as Clostridium and Bacteroides [153]. In a study by Noto et al., elevated levels of 3-(3-hydroxyphenyl)-3-hydroxypropanoic acid, 3,4-dihydroxybutyric acid, phenylalanine, tyrosine, *p*-hydroxyphenylacetic acid and homovanillic acid, the latter four of which are involved in the tyrosine pathway leading to the neurotransmitter catecholamine, were found in the urine of 21 ASD children compared to their siblings [154].

In addition to the factors mentioned above, the alteration of neurotransmitters is another potential mechanism linking dysbiosis to the development of ASD. Hyposerotonemia has been reported in the brains and blood of individuals with ASD [140,171]. According to some authors, the disruption of this biomarker could relate to intestinal dysbiosis because, as mentioned earlier, 95% of circulating serotonin is produced from intestinal enterochromaffin cells [85]. According to DeTheije et al., the low-grade inflammatory gut state of ASD children may induce enterochromaffin cells, mast cells and platelets to produce serotonin synthesis, inducing intestinal dysmotility and tryptophan consumption [172]. Moreover, dysbiosis can reduce tryptophan availability by decreasing amino acid uptake from the diet [173]. Consequently, despite higher levels of circulating serotonin from the gut, the effective availability of this neurotransmitter to the CNS may actually be decreased. Unfortunately, neither tryptophan supplementation nor selective serotonin reuptake inhibitors have ever proved effective [50].

There is still debate on the exact function of GM in ASD, and despite the opposing results of some studies, the comprehensive assessment of microbiome modifications due to both genetic and environmental influences could be useful in planning preventive strategies that act on the microbiota.

## 5. Alterations in Microbial Composition in ASD

There are many reasons why research has focused on alterations in GM as a risk factor for the development of ASD symptoms. For example, in a 2019 study by Sharon et al., it was found that germ-free mice exhibit behavior that resembles ASD after colonization with the fecal microbiota of human donors with autism [70]. In this study, subjects with ASD had a different abundance of *Clostridiaceae*, *Lactobacillales*, *Enterobacteriaceae* and *Bacteroides* compared to the control group. They also remarked that mice treated with the GM of ASD patients present alternative splicing of many ASD-related genes in the brain. In addition, a reduction in some metabolite profiles was observed in the ASD group, particularly 5-aminovaleric acid and taurine [70].

Bacterial species composition differs widely among individuals [174], although the microbiota of the GI tract is fairly preserved at the phylum level, where *Firmicutes* and *Bacteroidetes* prevail [174,175]. Many studies use fecal samples as a noninvasive proxy for GM because of the difficulties of sampling gut tissue (e.g., via colonoscopy). Although fecal microbiota seems to be of high representation in the colonic biota, it is a poorer proxy for other areas of the GI tract [176,177]. Stool sampling is still the preferred method for most studies of the intestinal microbiome in humans because fecal material comprises a subgroup of microorganisms that inhabit both the lumen and the mucosa.

Next-generation sequencing technologies through amplification, specifically by the PCR of the 16S ribosomal RNA gene for taxonomic classification, have contributed a culture-independent and very efficient approach for studying GM.

It is known that patients with ASD, compared with neurotypical children, largely present an altered intestinal microbial composition [178]. However, no particular microbial strains that might be associated with changes in various aspects such as age, diet, sex, population and ASD severity have been identified to be altered in all microbiota studies on ASD [179,180]. Patients with ASD often have microbial imbalances of various types, even though alterations in GM in patients with autism are not always the same across studies.

A commonly noticed phenomenon in the feces of ASD children is a reduced ratio of Bacteroidetes and Firmicutes, which might result from a reduction in the relative abundance of the phylum Bacteroidetes, responsible for digesting polysaccharides [114,180,181,182,183]. Therefore, Settanni et al. supported the hypothesis that people with ASD have abnormal carbohydrate digestion and intestinal mucosal dysbiosis [179,184]. Conflicting data were reported in the study conducted by De Angelis et al. on 30 subject children who reported higher numbers of Bacteroidetes than Firmicutes in ASD compared with those of the controls [185]. However, these findings were not statistically relevant [185]. In the same study, the phyla *Verrucomicrobia* and *Fusobacteria* were represented in lower concentrations in the feces of ten ASD compared to ten controls [185]. The significantly increased bacteria in ASD were *Akkermansia* spp., *Anaerophilum*, *Dorea* spp., *Clostridium* spp., *Barnesiella intestinihominis*, *Enterobacteriaceae*, *Roseburia* spp., *Faecalibacterium* spp. (especially *Faecalibacterium prausnitzii*), *Parasutterella excrementihominis*, *Turicibacter* spp., *Pseudomonas*, *Prevotella oris* and *Prevotella copri*. The significantly reduced bacteria in ASD were *E. coli*, *Bifidobacterium*, *Oscillospira*, *Fusobacterium*, *Subdoligranulum* spp. and *Streptococcus* spp. The least represented strains in children with ASD were *Lactobacillus*, *Lactococcus*, *Enterococcus* spp., *Collinsella* spp. (except *Collinsella aerofaciens*) and *Staphylococcus* [185].

In a study by Kang et al., the fecal microbiota of twenty autistic children with GI problems was compared with that of twenty neurotypical individuals. It showed significantly lower bacterial diversity in children with ASD [122,186]. In addition, they reported a decreased abundance of the *Coprococcus*, *Prevotellaceae* and *Veillonellaceae* in patients with autism [187]. *Prevotella* spp. are commensal intestinal microbes specialized in vitamin B1 synthesis and the degradation of vegetable polysaccharides. Thus, a vitamin B1 deficiency could be determined by a lower abundance of *Prevotella* spp. Minor differences have been observed in the genus *Sutterella*, which is less represented in ASD children [122]. These results suggest a changed composition of the microbiota in children with ASD that potentially could have biochemical and functional implications for the guest.

In earlier research, some *Prevotella* spp. (*P. oralis*, *P. ruminicola*, *P. tanneries*) were found to be reduced in patients with irritable bowel syndrome [36]. This research suggests that GI symptoms in ASD are related to altered balance in the microbiota [187].

In other past research, the amount of the genus *Akkermansia* was reported to decrease in children with ASD, whereas the amount of *Desulfovibrio* spp. increased [135,182]. The latter bacteria are recognized as being harmful, as they might exacerbate ASD patterns and GI disorders [188].

In a recent systematic review and meta-analysis conducted by Iglesias-Vázquez et al., including 18 studies with 493 autistic children and 404 healthy controls, it was found that GM is composed primarily of the genera Bacteroidetes, Actinobacteria and *Firmicutes*, all of which are more numerous in autistic children than they are in healthy controls [189].

Autistic children displayed a significantly increased abundance of the genera *Bacteroides*, *Phascolarctobacterium*, *Clostridium*, *Parabacteroides* and *Faecalibacterium* and minor amount of *Coprococcus* and *Bifidobacterium*. The authors concluded by suggesting the actual existence of dysbiosis in autistic children that could influence the development and severity of autistic symptomatology [189]. However, controversial results have been reported among the studies included in the analysis. For *Bacteroidetes*, most studies identified a higher abundance in autistic children [21,122,179,190,191,192], whereas others reported opposite results [180,193,194]; for *Firmicutes*, some studies have shown greater percentages in children with ASD [180,193,194], whereas others have shown greater percentages in healthy controls [21,190,192], with others even indicating no distinction between the groups [122,191]. For Actinobacteria, the relative abundance was not significantly different between those with ASD and the controls.

In a study by William et al., the microbiota was analyzed on intestinal biopsies of the cecum and terminal ileum, and significantly more *Sutterella* spp. were found in twenty-three children with ASD compared to those in nine controls. *Sutterella* spp. are usually poor in healthy GM and were not detected in any of the healthy controls. However, they were found in 12 of the 23 individuals with ASD [195]. Kushak et al. also studied microbial composition in duodenal biopsies comparing 19 ASD children with 21 controls without finding significant differences between phyla, but the authors observed some alterations at the genera and strain levels [196]. In particular, *Burkholderia* spp. was significantly augmented in subjects with ASD compared with the controls. *Oscillospira*, *Actinomyces*, *Ralstonia* and *Peptostreptococcus* were increased, and the genera *Bacteroides*, *Devosia*, *Neisseria*, *Prevotella* and *Streptococcus* were underrepresented in autistic children compared with the controls. In addition, lower abundances of Escherichia coli were reported [196].

Kushak et al. were unable to identify significant differences at the phylum level, as other research has cited [180,182,183,185], likely because all other studies analyzed the GM composition of fecal specimens and not duodenal mucosal biopsies. As previously mentioned, stool samples broadly represent the GM composition of the large bowel [197]. Kushak et al. instead mapped the GM composition of the small bowel, and in their conclusions, the authors suggested that microbial alterations in the large gut may have a more important effect on ASD than do alterations in the small bowel. Hence, a clearer portrayal of the GM environment may be obtained by collecting mucosal specimens from throughout the gut tract to confront the microbiota and better understand the interrelationships of GM functioning and interaction with the host.

A decreased amount of *Bacteroides*, with a significantly increased *Firmicutes*/*Bacteroidetes* ratio, was found in the ileum and cecum biopsies of 15 autistic children (7 controls) [183]. Firmicutes amounts were slightly increased in the autistic group. The *Betaproteobacteria* (Proteobacteria) class was significantly more copious in the fecal specimens from the cecum of autistic patients [183].

In addition, sibling control studies in the literature have led to discordant results.

In a Slovak study conducted by Tomova et al. on 29 participants, including ASD children, their siblings and healthy controls, they reported that the amounts of *Lactobacillus* spp., *Desulfovibrio* spp. and *Clostridia* spp. were significantly increased in the stools of subjects with ASD compared to those of their siblings and the controls. Moreover, *Bifidobacterium* spp. was decreased in autistic children [182]. *Desulfovibrio* spp. could be an essential factor in GI inflammation because its main metabolic byproduct, hydrogen sulfide, is cytotoxic to colonicmucosa [198]. In addition, when administered to rodents, *Desulfovibrio* spp. reduces working memory [199].

*Desulfovibrio* spp. abundance has been associated by researchers with the severity of behavioral symptoms in communication, social interaction and restricted/repetitive behavior in autism. In non-autistic siblings of children with ASD, a lower abundance of Bacteroidetes and *Bifidobacterium* spp. and a higher amount of *Desulfovibrio* spp. were shown than those in autistic children. Therefore, the authors hypothesized that the levels of bacterial strains may be the key turning factor between healthy and ASD phenotypes [182].

In contrast, no significant differences were found in a study conducted by Gondalia et al. on the microbial composition of 51 subjects with ASD and their 53 nonautistic siblings [193]. Likewise, another study that included 59 ASD children and 44 nonautistic siblings also reported no significant differences in GM composition [200]. These findings imply that siblings’ microbiota are similar and independent of their autism phenotype [193], likely due to the shared environment and genetic inheritance [56].

Compared with neurotypical healthy subjects, autistic children have been demonstrated to have a significantly higher amount of *Clostridium* spp., which has been associated with disease severity according to the Childhood Autism Rating score (CARs score) [178,201,202]. In a study conducted by Finnegold et al. on fecal samples from 33 autistic children, they observed a significantly higher rate of *Clostridium perfringens* producing beta2-toxin gene in ASD children compared to that in 13 controls [186]. Similarly, Alshammari et al. observed a greater presence of *Clostridium perfringens* in ASD children than that in the controls [203,204]. In addition, Beta2 toxin has been linked to more GI abnormalities, such as food poisoning and diarrhea [181]. Fisher et al. found that stool samples from patients with ASD had 79% beta2 toxin, whereas it was only 38% in the controls [181].

In a review, the authors suggested that a subacute tetanic infection with *Clostridium tetani*, an opportunistic pathogen, could cause some cases of ASD following the production of tetanus neurotoxin [94]. It inhibits the discharge of synaptic vesicles holding neurotransmitters by irreversibly cleaving synaptobrevin. Synapses with cleaved synaptobrevin degrade, and the decreased synaptic activity is correlated with the decreased social behavior found in ASD [94].

In addition, fungal gut dysbiosis has been found in the autistic population in addition to bacterial changes. The yeast *Candida albicans*, which makes ammonia and other toxins likely associated with ASD-related behavior, has been identified in the guts of autistic children more often than in those of non-ASD children [201,205]. A study by Kantarcioglu et al. found a higher amount of *Candida* spp. in the intestines of 415 autistic individuals compared with that in 403 controls [205]. The authors estimated that about 60% of the healthy population are asymptomatic carriers of *Candida* spp., which appears to be associated with some autistic behaviors [205]. In ASD subjects, altered microbiota diversity promotes the rise of *Candida* spp., which, once settled in the intestine, prevents the recolonization of commensal microbiota [180]. *Candida* spp. produces ammonia and toxins and also causes the malabsorption of minerals and carbohydrates, which could play a role in the pathophysiology of ASD [205].

All the cited studies have reportedd a dysbiotic bacterial profile in ASD children. The direction of causation, i.e., whether alterations in microbial profiles are related to the onset of ASD, is still under study.

## 6. Role of Probiotics in ASD

Evidence of the beneficial effects of probiotic and symbiotic formulations in individuals with ASD has been evaluated over the years through numerous studies (Table 2). Supplementation with probiotics allows for the use of an easy and noninvasive approach in modulating the composition and functions of GM and likely also in affecting host immune signaling, resulting in improved or prevented inflammation [187,206].

In a recent study conducted in an animal model of obsessive-compulsive disorder, the administration of *L. casei* Shirota reduced obsessive-compulsive disorder symptoms, which are likely secondary to the regulation of serotonin-related gene expression [216]. In another mouse model study, treatment with *Bacteroides fragilis* effectively reduced gut permeability and altered levels of tight junction proteins and cytokines, proving to be an attractive treatment for improving a leaky gut and behavioral symptoms [107].

In a randomized placebo-controlled trial conducted by Parracho et al., supplementation with *L. plantarum* WCSF1 for three months in children with ASD resulted in an amelioration of gut symptoms (*p* < 0.01) but also, more importantly, in an improvement in behavioral scores (*p* < 0.05) [207]. The authors observed an augmentation in enterococci and lactobacilli and a decrease in *Clostridium* cluster XIVa compared with those in the placebo group [207].

Kałużna-Czaplińska and Blaszczyk performed an open-label study by supplementing 22 ASD children aged 4 to 11 years with *L. acidophilus* Rosell-11 for two months [208]. The authors found that probiotic supplementation helps to mitigate behavioral issues that are characteristic of ASD and significantly reduce urinary levels of D-arabinitol, a metabolite of *Candida* spp., compared with the baseline (*p* < 0.05) [208].

West et al. examined the efficacy of Delpro^®^, a probiotic supplement with a mixture of five strains of *bifidobacterial* and *lactobacilli* with the addition of the immunomodulator Del-Immune V^®^ (*L. rhamnosus* V, cell lysate) in children with ASD (*n* = 33, 3–16 years) [210]. After three weeks of treatment, participants reported a 48% reduction in diarrhea severity and a 52% reduction in constipation severity [210]. In addition, 88% of the children showed an improvement in ASD symptoms with a decrease in ASD severity scores [210].

Another pilot study by Tomova et al. treated ASD children (*n* = 10, 5–17 years), their siblings (*n* = 9) and neurotypical controls (*n* = 10) with a mixture of probiotics (*Bifidobacterium*, *Lactobacillus* and *Streptococcus* spp.) for four months [182]. The researchers described a significant reduction in *Bifidobacterium* and *Desulfovibrio* spp. and in the normalization of the *Bacteroidetes*/*Firmicutes* ratio in the stools of children with ASD, but also a reduction in TNF-α levels, a key inflammatory cytokine in intestinal inflammation (*p* < 0.05) [182].

Treating ASD children (*n* = 30, 5–9 years old) for three months with a mixture of probiotics (*L. acidophilus*, *L. rhamnosus*, and *Bifidobacterium longum*), Shaaban et al. found a significant increase in *Bifidobacteria* and *Lactobacilli* in stool samples along with a reduction in autism severity scores compared to baseline results (*p* = 0.0001) and total GI symptom severity (*p* < 0.0001) [211].

A more recent cross-over, a randomized placebo-controlled pilot study by Arnold et al., evaluated the magnitude of the effect and safety of VISBIOME (formerly VSL#3), a mixture of eight probiotic strains of *Lactobacilli*, *Bifidobacteria* and *Streptococcus thermophiles* in children with ASD and comorbidities of anxiety and GI disorders (*n* = 13, 3–12 years) [212]. Probiotic supplementation produced nonsignificant and temporary improvements in GI symptoms compared with the placebo (*p* = 0.02) and did not change bacterial species diversity and microbiome composition [212].

Also, in another recent randomized double-blind placebo-controlled clinical trial conducted by Santocchi et al., VISBIOME was administered in preschool children with ASD (*n* = 63, 18–72 months) for six months [213]. Probiotic treatment did not lead to significant differences in autism severity or in fecal or blood inflammatory markers between groups [213]. In the subgroup analysis of patients without GI symptoms, probiotic supplementation significantly reduced total autism severity scores compared with the placebo (*p* = 0.026). Similarly, in the subgroup of patients with GI manifestations, there was a significant decrease in GI symptom severity scores compared with the placebo (*p* = 0.0191) [213].

Furthermore, if probiotics are used together with prebiotics, a substrate selectively used by host microorganisms that confers a health benefit, synbiotics are created, which are a mixture that includes live microorganisms and substrates selectively used by host microbiota that confer a health benefit to the host [217].

In a randomized clinical trial conducted by Sanctuary et al., children with ASD and GI comorbidities such as chronic constipation, diarrhea and/or irritable bowel syndrome (*n* = 8, 2–11 years) were randomized to receive *Bifidobacterium infantis* UCD272 in combination with a bovine colostrum product (BCP), a prebiotic or BCP alone [214]. Subjects had both treatments for five weeks, but in a different order according to randomization with a 2-week washout period. These supplementations showed that both were well tolerated by the patients; both treatments significantly reduced abdominal pain; a significant reduction in the incidence of diarrhea was only found in the BCP arm (*p* = 0.021); there was a decrease in the total aberrant behavior scores in the BCP group (*p* = 0.006); and there were no overall alterations in the GM or associated metabolites (fecal, urinary or blood) in either treatment arm [214]. According to researchers, an improvement in GI symptoms may be due to a significant reduction in the percentage of helper T lymphocytes (CD4+ T cells) expressing IL-13, a cytokine involved in allergic disorders, after treatment with a symbiotic, and in the percentage of CD8+ T cells expressing TNF-α (a cytokine involved in inflammatory responses) after treatment with BCP alone observed in the analysis of blood samples after treatment [214]. However, this study had a significant limitation determined by the low number of patients and the lack of a placebo arm [214].

In a 2020 randomized controlled trial, Wang et al. administered a probiotic mixture (one packet per day containing *L. rhamnosus* HN001, *Bifidobacterium lactis* BL-04, *Bifidobacterium infantis* Bi-26 and *L. paracasei* LPC-37) in combination with a prebiotic consisting of fructo-oligosaccharides (FOS) or a placebo in ASD children (*n* = 26, 2–8 years) for 30–108 days [215]. Supplementation showed a significant improvement in GI manifestations (diarrhea, constipation and fecal odor) in the treatment arm (*p* < 0.001), significantly reduced autism severity scores (*p* = 0.009), increased levels of *Bifidobacterium longum* (*p* < 0.001) and reduced levels of potentially pathogenic species, such as *Clostridium* spp. (*p* < 0.05) [215]. In addition, the normalization of blood serotonin and fecal SCFAs levels and decreased blood levels of zonulin (a marker of intestinal permeability) were observed (*p* < 0.01) [215].

Finally, in a study conducted by Pärtty et al., supplementation with *L. rhamnosus* GG was performed for its potential preventive role in neurodevelopmental diseases [209]. Seventy-five infants were randomized to receive the probiotic or placebo for 6 months with a 13-year follow-up. The researchers found that, after 13 years of follow-up, no children in the probiotic arm developed Asperger’s syndrome or attention-deficit/hyperactivity disorder (ADHD) (0%) compared with 17.1% in the placebo arm (*p* = 0.008). In addition, children with neurological impairment significantly showed a minor abundance of fecal *Bifidobacteria* in the first six months of life compared to healthy children (*p* = 0.03) [209].

In a 2019 systematic review conducted by Qin Xiang et al. on the effects of prebiotics/probiotics on ASD and its associated symptoms, the authors stated that the literature supporting the role of probiotics in alleviating GI or behavioral symptoms in ASD individuals is limited because there is no standardized probiotic regimen because of various species, concentrations and varying durations of supplementation [218].

Preliminary results on probiotic supplementation in ASD suggest that it is a promising approach to decrease the severity of ASD symptoms and to address GI comorbidities by exploiting probiotics’ capability to decrease the inflammatory potential of GM.

Nevertheless, given the heterogeneity of clinical trials, the possibility of producing an evidence base for targeted treatments is limited to date.

## 7. Conclusions

ASD is a complex disease that is typical of early age-related brain development that affects the quality of life of not only patients but also caregivers. The etiology of ASD remains unknown, but several factors may contribute to its onset, including genetic variations and environmental influences, especially during the peri-natal period. Scientific evidence in recent years has attributed a key role to GM in the pathogenesis of ASD, as it influences the development of the CNS and neuropsychological and GI homeostasis through the microbiota–gut–brain axis. Indeed, a state of dysbiosis with a reduction in the *Bacteroidetes*/*Firmicutes* ratio and the level of *Bacteroidetes* is common in the ASD community. Not surprisingly, there is a high incidence of functional GI disorders, especially diarrhea, chronic constipation and irritable bowel syndrome, which are often associated with dysbiotic states and a “leaky gut” in ASD subjects. In recent decades, many authors have attempted to study and identify the microbial signature on intestinal mucosa biopsies and fecal samples in ASDs through in vivo and ex vivo studies. In this regard, the advent of metabolomics has also been of great support. Based on these data, various therapeutic strategies, primarily probiotics, have been investigated to improve ASD symptoms through microbiota modulation. However, the heterogeneity of the studies precludes concrete evidence, although preliminary results are promising. To date, a holistic approach with a multidisciplinary team appears to be the winning weapon for the management of patients with ASD in clinical practice.

## Figures and Tables

**Table 1:** Metabolites in blood samples of ASD subjects from metabolomic studies.

References	N of Subjects	Analytical Technique	Increased Metabolites	Decreased Metabolites	Metabolic Process Involved
Kuwabara et al. (2013) [155]	32 ASD 40 controls	CE–TOF-MS	Taurine, arginine	Lactic acid, 5-oxoproline	Oxidative stress Mitochondrial dysfunction
West et al. (2014) [156]	52 ASD 30 controls	LC–MS and GC–MS	Aspartate, glutamate, DHEA sulfate, serine, glutaric acid, 5-aminovaleric acid lactam, 5-hydroxynorvaline, succinin acid, 3-aminoisobutyric acid, 2-hydroxyvaleric acid	Homocitrulline, lactic acid, 2-hydroxyvaleric acid, cystine, myristic acid, isoleucine, creatinine, methylhexadecanoic acid, 4-hydroxyphenyllactic acid, citric acid, heptadecanoic acid	Oxidative stress Mitochondrial disease or dysfunction Energy production Homocitrulline metabolism
Wang et al. (2016) [157]	173 ASD 163 controls	UPLC/Q-TOF MS/MS	Phytosphingosine, sphingosine 1-phosphate, pregnanetriol, LysoPC(20:3(5Z,8Z,11Z)), LysoPC(18:3(6Z,9Z,12Z)), 9,10-epoxyoctadecenoic acid	L-acetylcarnitine, decanoylcarnitine, adrenic acid, uric acid, arachidonic acid, docosahexaenoic acid, docosapentaenoic acid	Mitochondrial dysfunction Fatty acid β-oxidation
Anwar et al. (2018) [158]	38 ASD 31 controls	LC–MS	Nε-carboxymethyl-lysine, threonine, hydroimidazolone age derived from methylglyoxal, arginine, glutamine, glutamicacid, nω-carboxymethylarginine, glutamic semialdehyde, α-aminoadipic semialdehyde	3-deoxyglucosone, tryptophan, nε-fructosyl-lysine, n-formylkynurenine, hydroimidazolone age derived from glyoxal	Glucose glycation Oxidative stress Protein glycation
Barone et al. (2018) [159]	83 ASD 79 controls	ESI-Tandem MS/MS	Hexadecenoylcarnitine, octadecenoylcarnitine, acetylcarnitine, methylmalonyl/3-OH- isovalerylcarnitine, citrulline, decanoylcarnitine, tetradecadienoylcarnitine, dodecanoylcarnitine, hexadecanoylcarnitine		Biosynthesis of amino acids Fatty acid metabolism Mitochondrial dysfunction Gastrointestinal disturbances
Delaye et al. (2018) [160]	22 ASD 29 controls	Amino acid chromatographs		Glutamate, glycine, serine	Neurotransmitter metabolism
Grayaa et al. (2018) [161]	36 ASD 38 controls	GC–MS	24-Hydroxycholesterol	25-Hydroxycholesterol, 7α-hydroxycholesterol	Cytotoxicity Cholesterol metabolism Apoptosis Synaptic dysfunction Oxidative stress
Karhson et al. (2018) [162]	59 ASD 53 controls	LC–MS/MS		Anandamide	Retrograde endocannabinoid signaling
Lv et al. (2018) [163]	60 ASD 30 controls	MS/MS		Carnosyl carnitin, free carnitine, twenty-four carbonyl carnitine, octyl carnitine, glutaryl carnitine	Mitochondrial dysfunction Fatty acid metabolism
Aran et al. (2019) [164]	93 ASD 93 controls	LC–MS/MS		*N*-arachidonoylethanolamine, n-oleoylethanolamine, n-palmitoylethanolamine,	Retrograde endocannabinoid signaling
Kelly et al. (2019) [165]	403 children including 52 ASD	UPLC-MS/MS	Trimethylamine *N*-oxide, cinnamoylglycine, oleoyl ethanolamide, linoleoyl ethanolamide, docosahexaenoylcarnitine, prolylhydroxyproline, alpha-ketobutyrate, palmitoyl ethanolamide, erythritol, serotonin	*N*-formylphenylalanine, 5-hydroxyindoleacetate, pyrraline, n-formylanthranilic acid, sphingomyelin (d18:1/25:0, d19:0/24:1, d20:1/23:0, d19:1/24:0)	Endocannabinoid metabolism Xenobiotics and tryptophan metabolism Amino acid mebolism Phospholipid metabolism Fatty acid metabolism Urea cycle Sphingolipid metabolism
Orozco et al. (2019) [166]	167 ASD 193 controls	H-NMR	Glycine, serine, ornithine, cis-aconitate		Carbon metabolism TCA cycle Urea cycle
Rangel-Huerta et al. (2019) [167]	20 ASD 30 controls	UPLC–MS/MS	1-methylnicotinamide, 3-Indoxyl sulfate, 4-methyl-2-oxopetane, 5-bromotryptophan, sebacate, dodecanedioate, aspartate, orotate, galactitol, *N*-acetyl-aspartyl, 6-hydroxyindole sulfate, cortisone, methionine, tryptophan, arginine -glutamylmethionine, ursodeoxycholate, sphingomyelins, kynurenine, choline phosphate, decanoylcarnitine, 2-keto-3-deoxyglutamate, arachidate, behenate, fructose	Glutamate, 1-stearoyl-glycerol-phosphatidyl-etholamine, 1-palmitoyl-glycerol-phosphatidyl-etholamine, nicotinamide	Amino acid, lipid and nicotinamide metabolism Neurotransmitter metabolism
Smith et al. (2019) [168]	516 ASD 164 controls	Triple Quadrupole LC–MS	Glutamine, ornithine, glycine, ornithine to isoleucine ratio, ornithine to valine ratio, glutamine to leucine ratio, glycine to leucine ratio, glutamine to isoleucine ratio, ornithine to leucine ratio, glutamine to valine ratioglycine to isoleucine ratio, glycine to valine ratio	Isoleucine, leucine, valine	Amino acid metabolism Neurological impairments
Shen et al. (2022) [169]	5 ASD 5 controls	UPLC/Q-TOF MS/MS	L-Glutamine	L-Glutamate, alanine, aspartate, O-phospho-4-hydroxy-Lthreonine, pyridoxamine, 4-pyridoxate	Niacin and niacinamide metabolism Vitamin B6 metabolism Arginine biosynthesis Sphingolipid metabolism
Wang et al. (2022) [170]	29 ASD 30 controls	LC–MS/MS	Valine, palmitoleic acid, epsilon-caprolactam, arachidonic acid, prostaglandin D2	Choline, 5-aminoimidazole ribonucleotide, 1-acylglycerophosphocholine, deoxyribose, benzoic acid, 3-butynoate, ornithine	Arachidonic acid metabolism Serotonergic synapse Choline metabolism

ASD, Autism Spectrum Disorder; CE–TOF-MS, Capillary Electrophoresis–Time-Of-Flight Mass Spectrometry; LC–MS, Liquid Chromatography–Mass Spectrometry; GC–MS, Gas Chromatography–Mass Spectrometry; MS/MS Tandem Mass Spectrometry; UPLC/Q-TOF-MS Ultra-High Performance Liquid Chromatography–Quadrupole Time-Of-Flight Mass Spectrometry; H-NMR, Hydrogen-1 Nuclear Magnetic Resonance; TCA, Tricarboxylic Acid Cycle; DHEA, Dehydroepiandrosterone.

**Table 2:** Clinical studies of probiotic and synbiotic supplementation in children with ASD.

References	Study Type	Probiotic Strains (Doses)	Duration	Sample Size (N)	Study Population	Main Results
***Single strain***						
Parracho et al. (2010) [207]	Randomized, double-blind, placebo-controlled	*L. plantarum* WCSF1 (4.5 × 10^10^ CFU/capsule)	3 weeks	39	4–16-year-old children with ASD, UK	↑ Lactobacilli and Enterococci, ↓ *Clostridium* cluster XIVa in stools Improved total behavior scores (DBC-P scores) No major differences in GI symptoms Improved stool consistency
Kaluzna-Czaplinska et al. (2012) [208]	Prospective, open-label, no controls	*L. acidophilus* Rosell-11 (5 × 10^9^ CFU/capsule)	Twice daily for 2 months	22	4–10-year-old children with ASD, 90% male, Poland	↑ D-arabinitol urine levels (*p* < 0.05) before and after treatment ↓ D-arabinitol and the ratio of D-/L-arabinitol in urine Improvement in the ability to concentrate and carry out orders
Partty et al. (2015) [209]	Randomized, placebo-controlled	*L. rhamnosus* GG ATCC 53,103 (1 × 10^10^ CFU)	Once daily for 7 months (1 month pre-delivery and 6 months post) 13-year follow-up	75	Infant intervention (*n* = 40) Controls (*n* = 35), Finland	At 13 y, 17.1% of the placebo arm had ADHD or Asperger’s Syndrome or ADHD vs. 0% of individuals in the probiotic arm (*p* = 0.008) ↓ *Bifidobacterium spp.* in children with neurologica impairment at 6 months of age compared to controls (*p* = 0.03)
***Combinations***						
West et al. (2013) [210]	Prospective, open-label, no control	Delpro^®^: *L. acidophilus*, *L. casei*, *L. delbrueckii*, *Bifidobacterium longum*, *Bifidobacterium bifidum +* immunomodulator Del-Immune V^®^ (1 × 10^8^ CFU)	Three times daily for 3 weeks	33	3–16-year-old children with ASD + GI issues, USA	48% of participants ↓ diarrhea 52% of participants ↓constipation 88% of participants improved behavior (↓ ATEC score)
Tomova et al. (2015) [182]	Prospective, open-label, controlled	Three strains of *Lactobacillus*, two strains of *Bifidobacteria*, one strain of *Streptococcus*	Three times daily for 4 months	29	2–9-year-old children with ASD (*n* = 10), 5–17-year-old siblings of ASD children (*n* = 9), 2–11-year-old control children (*n* = 10), Slovakia	↓ Bifidobacteria and *Desulfovibrio* spp. in stools Normalization Bacterioides/Firmicutes ratio ↓ Fecal TNF-α
Shaaban et al. (2018) [211]	Prospective, open-label	*L. acidophilus*, *L. rhamnosus*, *Bifidobacteria longum* (100 × 10^6^ CFU)	Once daily for 3 months	30	5–9 year old children with ASD (*n* = 30, 63% male), Controls (*n* = 30, matched age/gender), Egypt	↑ Bifidobacteria and lactobacilli in stools Improved behavior (↓ ATEC scores) ↓ Total GI symptom severity (6-GSI scores)
Arnold et al. (2019) [212]	Randomized, placebo- controlled, cross-over pilot study	Visbiome^®^ (formerly VSL#3) *L. acidophilus*, *L. plantarum; L. para-casei*, *L. delbrueckii* subsp. *Bulgaricus*, *Streptococcus thermophiles*, *Bifidobacterium longum*, *Bifidobacterium breve*, *Bifidobacterium infantis* (90 × 10^10^ CFU/packet)	Twice a day for 8 weeks	13	3–12-year-old children with ASD + anxiety + FGID	PedsQL GI score temporarily improved (*p* = 0.14) ↓ Parent-targeted GI symptoms (*p* = 0.02) No shift in gut microbiome diversity or family level composition of species
Santocchi et al. (2020) [213]	Randomized, placebo-controlled	Visbiome^®^ (formerly VSL#3, for composition see above) (450 billion CFU/packet)	6 months: Two packets/day for 4 weeks, one packet/day for 5 months	63	18–72 month-old children with ASD (*n* = 31), Controls with ASD (*n* = 32, matched age/gender), Italy	No significant changes in ASD severity score (ADOS-CSS score) No significant difference in blood biomarkers (IL-6, TFN-α) or fecal calprotectin Subgroup analysis: - No GI group, *n* = 46 ↓ Total autistic severity scores (ADOS) (*p* = 0.026) - GI group, *n* = 17 ↓ GI severity total (*p* = 0.0191), ↓ stool smell (*p* < 0.001), and ↓ flatulence (*p* = 0.0187)
***Synbiotic***						
Sanctuary et al. (2019) [214]	Randomized, double-blind, controlled, cross-over	*Bifidobacterium infantis* UCD272 (2 × 10^9^ CFU/day) + BCP (0.15 g/lb body weight/day) vs. BCP alone (0.15 g/lb body weight/day)	Once daily for 5 weeks, 2-week washout, 5 weeks	8	2–11-year-old children with ASD + GI comorbidity, America	↓ GI pain in both group ↓ Frequency of diarrhea in BCP arm (*p* = 0.021) ↓ In total aberrant behavior scores in BCP only (*p* = 0.006) ↓ IL-13 (post symbiotic intervention, *p* = 0.006) and TNF-α release in stimulated cells
Wang et al. (2020) [215]	Randomized, placebo- controlled	*Bifidobacterium infantis* Bi-26, *L. rhamnosus* HN001, *Bifidobacterium lactis* BL-04 and *L. paracasei* LPC-37 + FOS	Once daily for 108 days (measures taken at 30, 60 and 108 days)	50	Phase 1: 2–8-year-old children with ASD (*n* = 26), Controls (*n* = 24, matched age/gender) Phase 2: RCT Intervention (*n* = 16), Controls (*n* = 10), China	Phase 2—probiotic + FOS intervention vs. placebo: ↓ in ASD severity scores (60 days, *p* = 0.04; 108 days, *p* = 0.009) ↓ in severity of GI symptoms (30 days, *p* = 0.002; 60 days *p* = 0.001; 108 days, *p* < 0.001) ↑ *Bifidobacterium longum* and ↓ *Ruminococcus* and *Clostridium* spp. ↑ SCFAs, ↓ plasma zonulin and ↓ plasma serotonin

ADOS, Autistic Symptoms and Subthreshold Score; ATEC, Autism Treatment Evaluation Checklist Structure; BCP, Bovine Colostrum Product; CFU, Colony Forming Unit; DBC, Developmental Behavior Checklist; FGID, Functional GI Disorders; FOS, fructo-oligosaccharide; GI, gastrointestinal; 6-GSI, 6-Item Gastrointestinal Severity Index; IL, interleukin; L., *Lactobacillus*; PedsQL, Pediatric Quality of Life Inventory; SCFAs, short chain fatty acids; TNF-α, tumor necrosis factor alpha; ^®^ registered trademark; ↑ increased; ↓ decreased.

## Data Availability

All data are presented within the manuscript’s text and tables.
